# Oseltamivir–Resistant Pandemic H1N1/2009 Influenza Virus Possesses Lower Transmissibility and Fitness in Ferrets

**DOI:** 10.1371/journal.ppat.1001022

**Published:** 2010-07-29

**Authors:** Susu Duan, David A. Boltz, Patrick Seiler, Jiang Li, Karoline Bragstad, Lars P. Nielsen, Richard J. Webby, Robert G. Webster, Elena A. Govorkova

**Affiliations:** 1 Division of Virology, Department of Infectious Diseases, St. Jude Children's Research Hospital, Memphis, Tennessee, United States of America; 2 Department of Pathology, University of Tennessee Health Science Center, Memphis, Tennessee, United States of America; 3 Hartwell Center for Bioinformatics and Biotechnology, St. Jude Children's Research Hospital, Memphis, Tennessee, United States of America; 4 National Influenza Laboratory, Department of Virology, Statens Serum Institute, Copenhagen, Denmark; National Institutes of Health, United States of America

## Abstract

The neuraminidase (NA) inhibitor oseltamivir offers an important immediate option for the control of influenza, and its clinical use has increased substantially during the recent H1N1 pandemic. In view of the high prevalence of oseltamivir-resistant seasonal H1N1 influenza viruses in 2007–2008, there is an urgent need to characterize the transmissibility and fitness of oseltamivir-resistant H1N1/2009 viruses, although resistant variants have been isolated at a low rate. Here we studied the transmissibility of a closely matched pair of pandemic H1N1/2009 clinical isolates, one oseltamivir-sensitive and one resistant, in the ferret model. The resistant H275Y mutant was derived from a patient on oseltamivir prophylaxis and was the first oseltamivir-resistant isolate of the pandemic virus. Full genome sequencing revealed that the pair of viruses differed only at NA amino acid position 275. We found that the oseltamivir-resistant H1N1/2009 virus was not transmitted efficiently in ferrets via respiratory droplets (0/2), while it retained efficient transmission via direct contact (2/2). The sensitive H1N1/2009 virus was efficiently transmitted via both routes (2/2 and 1/2, respectively). The wild-type H1N1/2009 and the resistant mutant appeared to cause a similar disease course in ferrets without apparent attenuation of clinical signs. We compared viral fitness within the host by co-infecting a ferret with oseltamivir-sensitive and -resistant H1N1/2009 viruses and found that the resistant virus showed less growth capability (fitness). The NA of the resistant virus showed reduced substrate-binding affinity and catalytic activity *in vitro* and delayed initial growth in MDCK and MDCK-SIAT1 cells. These findings may in part explain its less efficient transmission. The fact that the oseltamivir-resistant H1N1/2009 virus retained efficient transmission through direct contact underlines the necessity of continuous monitoring of drug resistance and characterization of possible evolving viral proteins during the pandemic.

## Author Summary

Most of the currently circulating pandemic H1N1/2009 (“swine”) influenza viruses are susceptible to the anti-influenza drug oseltamivir. Many countries have stockpiled oseltamivir for pandemic preparedness, and to date only a small proportion of the H1N1/2009 viruses isolated have been oseltamivir-resistant. However, if these viruses can be readily transmitted, oseltamivir resistance may spread. We evaluated the transmissibility of a pair of pandemic H1N1/2009 influenza viruses in ferrets. One virus was oseltamivir-sensitive and the other carried the oseltamivir resistance-associated H275Y NA mutation. We also investigated the viruses' susceptibility to NA inhibitors (the drug class to which oseltamivir belongs), their NA enzyme kinetics, and their replication efficiency in cultured cells. Under identical conditions, the resistant H1N1/2009 virus was not transmitted by respiratory droplets but was efficiently transmitted by direct contact, while the sensitive H1N1/2009 virus was efficiently transmitted by both routes.

## Introduction

A novel swine-origin H1N1 influenza virus emerged in Mexico in April 2009 and rapidly spread worldwide, causing the first influenza pandemic of the 21st century [1], [2]. Most confirmed human cases of H1N1/2009 influenza have been uncomplicated and mild [3], but the increasing number of cases and affected countries warrant optimal prevention and treatment measures. At present, two classes of antiviral drugs are approved for specific management of influenza: M2-ion channel blockers (amantadine and rimantadine) and neuraminidase (NA) inhibitors (zanamivir and oseltamivir). However, variants resistant to both classes of drugs have emerged. During the 2007–2008 season, most circulating seasonal H3N2 influenza viruses, and H1N1 viruses in certain geographic areas, were reportedly resistant to M2-blockers [4], [5]; today, almost all of the pandemic H1N1/2009 viruses tested are resistant to M2-blockers [6]. Therefore, only the NA inhibitors are currently recommended for treatment of influenza [7].

The NA-inhibitor resistance-associated mutations in influenza viruses are drug-specific and NA subtype-specific [8]. Until 2007, the clinical data indicated only sporadic, rare emergence of oseltamivir resistance under drug selection pressure (<1% in adults and 4%–8% in children) [9]–[11]. Later reports observed increased frequency of oseltamivir-resistant variants (18% and 27%) in drug-treated children [11], [12]. The situation changed dramatically during the 2007–2008 season, when seasonal H1N1 influenza viruses with the common oseltamivir-resistance NA H275Y mutation (275 in N1 numbering, 274 in N2 numbering) became widespread in first the northern [13] and then the southern [14] hemispheres. It remains uncertain where these naturally resistant H1N1 influenza viruses originated and how they acquired optimal fitness and transmissibility, but the resistant variants were clearly becoming the dominant strain at the time the swine-origin pandemic H1N1/2009 virus emerged [15]–[17].

During the H1N1/2009 influenza pandemic, to date, almost all tested viruses have remained susceptible to oseltamivir and zanamivir [6], but oseltamivir-resistant variants with H275Y NA mutation have been isolated from individuals receiving prophylaxis [18], [19] and from immunocompromised patients [20] under drug selection pressure. Oseltamivir-resistant variants also have been isolated from untreated patients [21], [22] and from a few community clusters [23]–[25], including two suspected cases of nosocomial transmission among immunocompromised patients [23], [24], although it is uncertain whether the mutants came from secondary transmission or arose spontaneously. The isolation of resistant H1N1/2009 viruses with no link to oseltamivir use raised serious concern that these viruses might acquire fitness and spread worldwide, as had oseltamivir-resistant seasonal H1N1 viruses during 2007–2008.

The increasing concern about oseltamivir-resistant H1N1/2009 viruses prompted us to evaluate transmissibility and growth fitness of one oseltamivir-resistant variant. The infectivity and transmissibility (and thus the clinical relevance) of several NA inhibitor-resistant influenza viruses have previously been studied in experimental animal models [26]–[29]. These studies differed in the influenza A subtypes studied (H1N1, H3N2, or H5N1), the NA mutations involved (H275Y, R292K, E119V or I222V), the animal model used (ferret or guinea pig), and the transmission routes studied (direct contact and respiratory droplets); in these studies, the transmissibility of most of the NA inhibitor-resistant influenza viruses was to some extent less efficient. Here we characterized *in vitro* and in a ferret model a pair of pandemic H1N1/2009 clinical isolates. The pandemic A/Denmark/524/09 (A/DM/524/09) and A/Denmark/528/09 (A/DM/528/09) viruses were isolated from a small cluster of patients with H1N1/2009 virus infection [30]. The A/DM/528/09 virus, carrying the H275Y NA mutation, was isolated from a patient on oseltamivir prophylaxis, and its ancestor is likely to have been A/DM/524/09 virus. By recapitulating two natural routes of influenza virus transmission in ferrets, we found that the oseltamivir-resistant virus was less transmissible than its sensitive counterpart through the respiratory droplet route but retained efficient transmission through direct contact.

## Results

### Sequencing and phylogenetic analysis of NA genes

Sequence analysis of the NA genes revealed that A/DM/524/09 virus encoded a conserved H residue at amino acid position 275, whereas A/DM/528/09 virus had an H275Y amino acid mutation caused by a single T-to-C nucleotide substitution at codon 275 (Table 1). Pairwise sequence analysis of the full viral genomes showed that the A/DM/524/09 and A/DM/528/09 viruses had no amino acid differences other than the H275Y NA mutation and were a highly matched pair. Sequence analysis and phylogenetic analysis of the two viruses' NA and HA genes (data not shown) confirmed that the wild-type A/DM/524/09 and mutant A/DM/528/09 viruses belonged to the swine-origin 2009 pandemic virus lineage. The alignment of the NA and HA sequences showed that viruses with H275Y NA substitution have some amino acid differences from certain wild-type viruses (without H275Y NA mutation), but these differences also were observed in other wild-type viruses. Comparison of the NA and HA amino acid sequences of A/DM/528/09 virus with sequences of other 24 H275Y mutants and around 2000 wild-type H1N1/2009 viruses available in Gene Bank did not reveal an increased frequency of any specific amino acid mutation(s) shared among the viruses analyzed (data not shown).

**Table 1:** Neuraminidase enzymatic properties of the H1N1 influenza viruses.

H1N1 virus	Sequence at NA position 275^a^		NA enzyme inhibition IC_50_ ±SD^b^ (nM)		Enzyme kinetics^c^	
	Nucleotide	Amino acid	Oseltamivir carboxylate	Zanamivir	Km (µM)	Vmax (U/sec)
A/Denmark/524/09	CAC	H	5.0±0.8	1.3±0.15	55.1±4.2	101.6±7.9
A/Denmark/528/09	TAC	Y	972±283*	1.0±0.13	80.3±6.0*	86.8±5.6*

a The full genomes of both viruses were sequenced; only differences are shown. In order of segments, the GenBank accession numbers are CY043339–CY043346 for A/DM/524/09 virus and CY043347–CY043354 for A/DM/528/09 virus genome sequences.

b Mean ± SD from five independent measurements.

c Assayed in parallel with reference A/Fukui/08/02 (H3N2) virus. Km and Vmax values were derived from the Michaelis-Menten plot.

*P<0.05 compared to value for respective wild-type virus.

### NA inhibitor susceptibility and NA enzyme kinetics

To assess the NA inhibitor susceptibility of the two viruses, we performed NA enzyme inhibition assays with the NA inhibitors oseltamivir carboxylate (active metabolite of oseltamivir) and zanamivir. The wild-type A/DM/524/09 virus was susceptible to oseltamivir carboxylate (mean IC_50_: 5.0 nM), but the A/DM/528/09 carrying the H275Y NA mutation had IC_50_ values approximately 200 times that of the wild-type virus (Table 1). The IC_50_ of zanamivir was comparable for both viruses and was uniformly low (mean IC_50_≤1.3 nM) (Table 1). These results showed that the H275Y NA mutation conferred resistance to oseltamivir carboxylate but did not alter susceptibility to zanamivir.

To understand the impact of the H275Y mutation on the NA enzymatic properties of the H1N1/2009 viruses, we determined the NA enzyme kinetics of both viruses. Km is an estimate of the dissociation equilibrium for substrate binding to enzyme and the reciprocal of Km approximates the affinity of substrate binding, while Vmax reflects the enzyme's catalytic activity. The NA of resistant A/DM/528/09 virus had a slightly higher Km and lower Vmax than the NA of the sensitive A/DM/524/09 virus (Table 1). The H275Y NA mutation reduced NA affinity for substrate and NA catalytic activity, although the function of NA was not severely impaired. This finding in the H1N1 pandemic virus is similar to that reported by another group, in which NA enzymatic function was not impaired in some naturally resistant seasonal viruses isolated during the 2007 season [31]. Our study is the first to show reduced but not severely impaired NA enzymatic function in a resistant H1N1/2009 virus with the H275Y mutation.

### Plaque morphology and growth kinetics in MDCK and MDCK-SIAT1 cells

**Figure 1:**
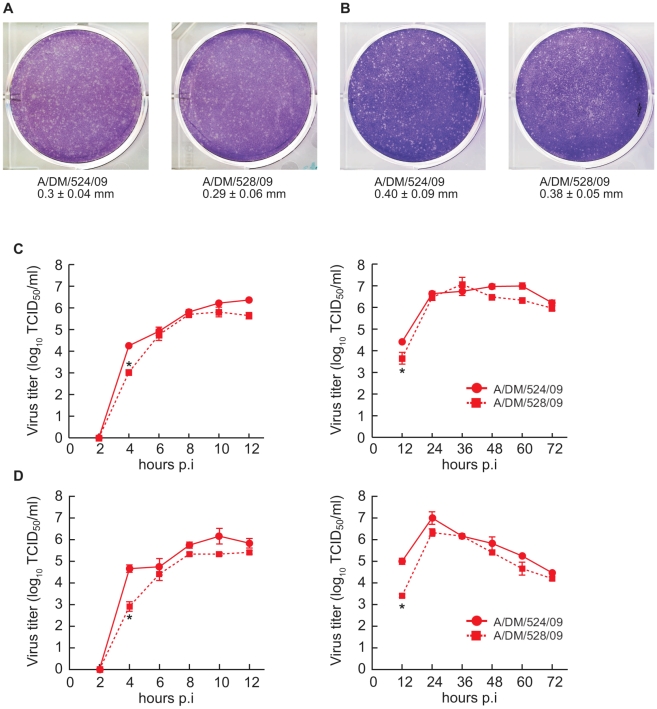
Plaque morphology and replication kinetics of two H1N1/2009 influenza viruses in MDCK and MDCK-SIAT1 cells. The diameters of 20 randomly selected value plaques were measured in MDCK cells (A) and MDCK-SIAT1 cells (B). Values are mean (± SD) plaque diameter (mm). Single-cycle (C, D left panel) and multiple-cycle (C, D right panel) growth curves were obtained by using an MOI of ∼2 and ∼0.001 PFU/cell, respectively. Virus in the supernatant was titrated in MDCK or MDCK-SIAT1 cells and expressed as log_10_TCID_50_/ml at the indicated time post-infection. Each point represents the mean log_10_TCID_50_/ml ± SD from three experiments. * P<0.05 compared to value for wild-type viruses.

To determine whether the H275Y NA mutation affects virus growth *in vitro*, we characterized virus plaque morphology and growth kinetics in both MDCK and MDCK-SIAT1 cells. The latter have increased surface expression of human-like α2,6-linked terminal sialic acids [32] and may better assess the growth capability of human influenza viruses. In MDCK cells, both pandemic H1N1/2009 viruses formed pinpoint-like (0.3 mm) plaque phenotype (Figure 1A), differing significantly from some seasonal H1N1 viruses, such as A/Brisbane/59/2007 (BR/59/07) virus, which formed large plaques (1.3 mm) (P<0.05) (data not shown); however, the plaque size did not differ between the oseltamivir-sensitive and -resistant viruses (Figure 1A), indicating that the H275Y NA mutation did not alter plaque morphology. In MDCK-SIAT1 cells, both the pandemic viruses and seasonal BR/59/07 (data not shown) formed only pinpoint-like plaques (Figure 1B), consistent with a previous report [32] that this cell line did not generate clear plaques for influenza viruses.

To further evaluate the impact of the H275Y NA mutation on virus growth *in vitro*, we performed single- and multiple-cycle growth studies of both viruses in MDCK and MDCK-SIAT1 cells. In single-cycle growth in the two cell lines, the two viruses reached comparable levels 6 hours post-infection, but the initial growth of the oseltamivir-resistant virus was significantly delayed in comparison to its sensitive counterpart (P<0.05) (Figure 1C): at 4 hours post-infection, the yield of resistant viruses was at least 1 log_10_TCID_50_/ml lower (P<0.05). Likewise, in multiple-cycle growth, the two viruses reached comparable yields 24 hours post-infection, but the resistant virus showed a significant growth delay during the first 12 hours post-infection (P<0.05); this delay was more conspicuous in MDCK-SIAT1 cells than in MDCK cells (Figure 1D), probably because overexpressed α2, 6 receptors on cell surface could better differentiate NA's function in support of viral growth. Therefore, final virus yields of oseltamivir-resistant pandemic virus in the MDCK and MDCK-SIAT1 cells were not altered, but their growth at the initial infection stage was significantly delayed.

### Transmissibility among ferrets via direct contact and respiratory droplets

The transmissibility of pandemic H1N1/2009 viruses was studied in a ferret model. Two naïve ferrets were housed at day 2 post-inoculation (p.i.) in the same cage with one inoculated ferret (direct contact), and two naïve ferrets were placed in an adjacent cage separated from the donor's cage by two layers of wire mesh (respiratory droplet exposure). Transmission of H1N1 virus was assessed by detection of infection in recipient ferrets (nasal wash titers, clinical signs, and seroconversion). Virus samples in nasal washes at day 4 p.i. or post-contact (p.c.) were sequenced to detect the presence of the H275Y NA mutation.

**Figure 2:**
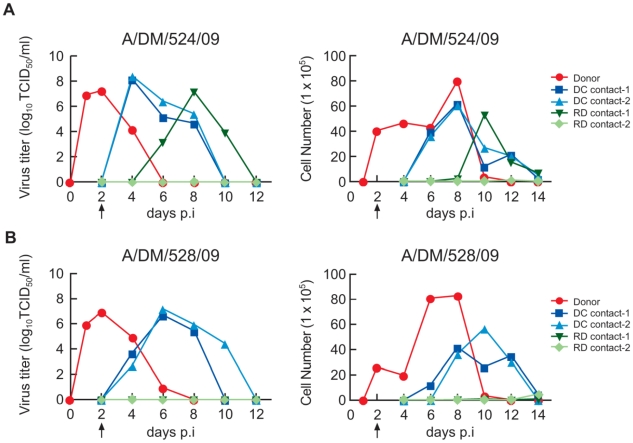
Transmissibility of the two H1N1/2009 influenza viruses among ferrets. The virus titer (A, B left panel) and total number of inflammatory cells (A, B right panel) in the nasal wash samples from each donor ferret, direct-contact (DC contact) ferret, and respiratory droplet-contact (RD contact ) ferret. The arrow indicates the first day of exposure of contact ferrets.

The donor ferret inoculated with oseltamivir-sensitive A/DM/524/09 virus shed virus until day 6 p.i. (Figure 2A, Table 2). Two of 2 direct-contact ferrets and 1 of 2 respiratory droplet-exposed ferrets were infected through virus transmission, as indicated by the virus titers and inflammatory cell counts in their nasal washes (Figure 2) and by seroconversion (Table 3). Virus shedding and nasal inflammation began earlier in the direct-contact ferrets, suggesting that transmission through respiratory-droplets may have a greater lag time. One respiratory droplet-exposed ferret showed no detectable virus shedding or inflammation, but its post-contact serum had a positive HI titer (320). Although seroconversion indicated infection in this ferret, the time of infection could not be determined and therefore we could not attribute the infection to direct contact with the co-caged ferret versus respiratory droplet transmission from the adjacent cage.

**Table 2:** Clinical signs, virus replication, and seroconversion in inoculated donor ferrets.

H1N1 virus	Inoculated donor ferrets^a^					
	Clinical signs			Virus replication		
	Weight loss^b^ (%)	Sneezing (observed day of onset)	Last day of shedding^e^	Peak virus titer (day p.i)^d^	Last day of shedding^e^	Serum HI titer^f^
A/Denmark/524/09	5.0 (2)	3	12	7.3 (2)	6	1280
A/Denmark/528/09	6.2 (2)	7	12	6.9 (2)	8	640
Co-inoculation^g^	5.9 (4)	7	12	7.7 (2)	6	640

a n = 1 for each virus group.

b The maximum percent weight loss during the 21 days p.i. Numbers in parentheses indicate the day of maximum weight loss.

c Upper respiratory tract inflammation was defined as a total inflammatory cell count ≥10 times the baseline count.

d Virus titers in nasal washes (log_10_TCID_50_/ml).

e The first day of observation on which virus was not detected.

f Hemagglutination inhibition (HI) antibody titers to homologous virus 21 days p.i.

g Co-inoculation of ferret with A/DM/524/09 and A/DM/528/09 viruses at 1∶1 ratio.

**Table 3:** Clinical signs, virus replication, and seroconversion in contact ferrets.

H1N1 virus	Direct contact					Respiratory droplets exposure				
	Clinical signs		Virus detection			Clinical signs		Virus detection		
	Weight loss^a^	Sneezing^b^	Virus shedding^c^	Last day of shedding^d^	Serum HI titer^e^	Weight loss^a^	Sneezing^b^	Virus shedding^c^	Last day of shedding^d^	Serum HI titer^e^
A/Denmark/524/09	2/2 (3.5)	1/2 (7)	2/2 (8.3)	8, 8	1280, 640	1/2 (6.0)	1/2 (7)	1/2 (7.2)	10	1280,320
A/Denmark/528/09	2/2 (3.3)	2/2 (5,7)	2/2 (7.0)	8, 10	1280,1280	0/2	0/2	0/2	NA	<10,<10
Co-inoculation^f^	2/2 (6.0)	1/2 (2)	2/2 (7.1)	10, 10	1280,1280	NA	NA	NA	NA	NA

a Number of animals with weight change/total number (maximum percent weight loss during the 21 days p.c.).

b Number of animals sneezing/total number during the 21 days p.c. (day of observed onset).

c Number of virus-shedding animals/total number. Numbers in parentheses indicate mean peak virus titer (log_10_TCID_50_/ml) in nasal wash samples).

d The first day of observation on which virus was not detected.

e Hemagglutination inhibition (HI) antibody titers to homologous virus in ferret serum on day 21 p.c.

f Donor ferret was co-inoculated with A/DM/524/09 and A/DM/528/09 viruses at a 1∶1 ratio.

The donor ferret inoculated with oseltamivir-resistant A/DM/528/09 virus shed virus until day 8 p.i. (Figure 2), with a peak virus titer comparable to that of A/DM/524/09 virus (Table 2). Two of 2 direct-contact ferrets were infected through transmission (Figure 2), but neither respiratory droplet-exposed ferret was infected, as confirmed by the absence of seroconversion (Table 3). These results showed that the oseltamivir-resistant H275Y mutant A/DM/528/09 virus was transmitted efficiently only by direct contact. Virus shedding in two direct-contact ferrets was lower and peaked after a longer interval in this group than in the oseltamivir-sensitive A/DM/524/09 group (Figure 2A), although the severity and course of disease were similar (Figure 2B, Table 3).

We verified the sequence stability of the NA at position 275 in each virus after replication and transmission in ferrets. Direct sequencing of the NA genes from nasal wash samples revealed no sequence change at this position in either virus (data not shown). Therefore, no spontaneous H275Y NA mutation emerged in the wild-type virus and the H275Y mutation remained stable in the mutant after transmission to a new host.

### Co-inoculation with oseltamivir-sensitive and -resistant H1N1/2009 viruses

**Figure 3:**
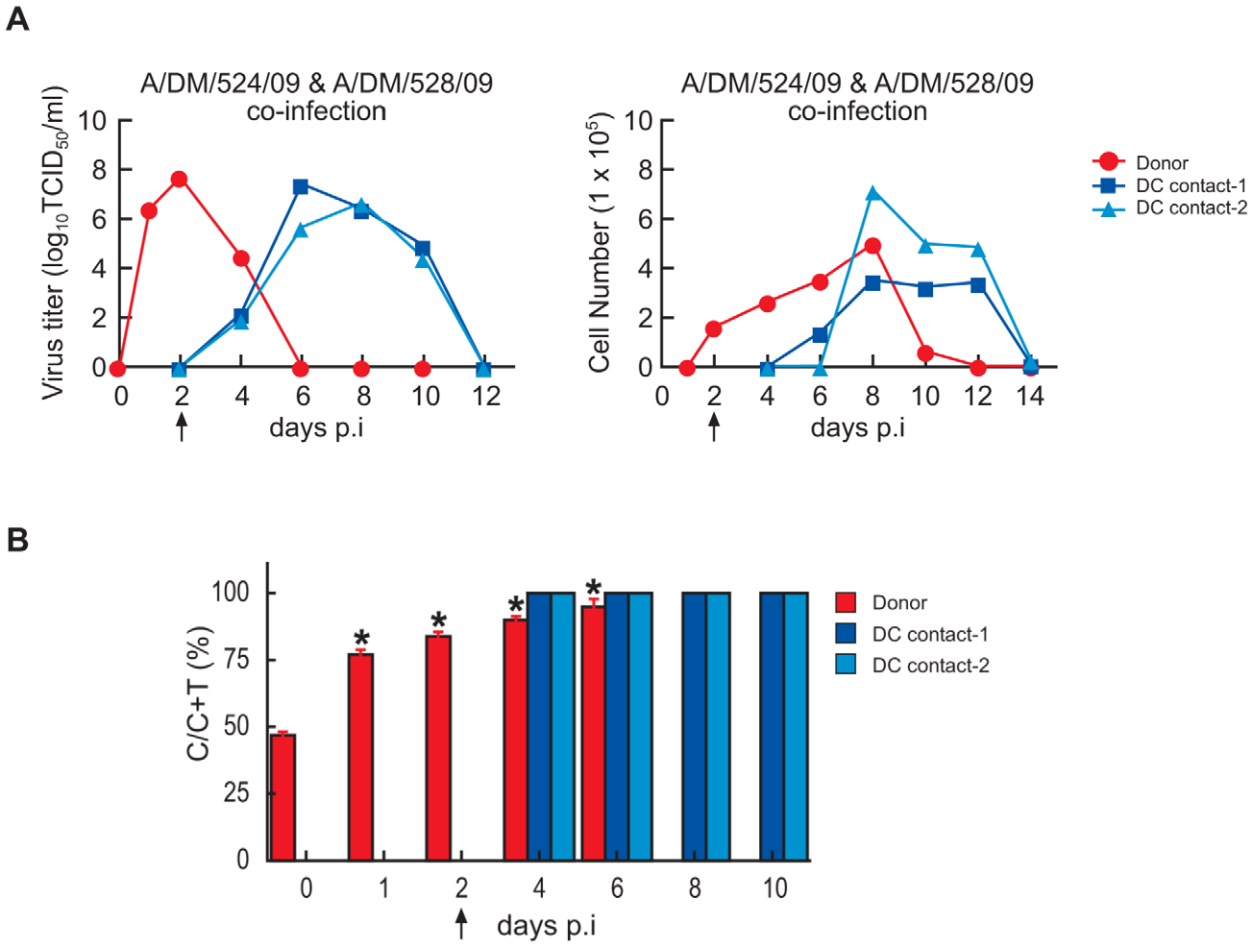
Co-infection in a ferret with oseltamivir-sensitive and -resistant H1N1/2009 influenza viruses. Virus titers and inflammatory cell counts in the nasal wash specimens of ferrets co-inoculated with oseltamivir-sensitive and -resistant H1N1/2009 viruses (A). The arrow indicates the first day of exposure of contact ferrets. The proportion of wild-type virus (C in SNP sequence) in the mixed virus population (C+T in SNP sequence) in nasal wash samples from the donor ferret and two direct-contact ferrets (B). Values are the mean ± SD from three independent measurements. * P<0.05 compared to value for day 0 p.i.

Because both the oseltamivir-sensitive and the oseltamivir-resistant H1N1/2009 viruses were efficiently transmitted by direct contact, hosts could potentially be exposed to both types of virus. To compare the relative growth capability and transmissibility of the sensitive and resistant H1N1/2009 viruses within the host, we co-inoculated a ferret with a 1∶1 ratio of the sensitive A/DM/524/09 and resistant A/DM/528/09 viruses. The pattern of virus shedding and the clinical signs were similar to those in ferrets inoculated with either A/DM/524/09 or A/DM/528/09 virus (Figure 3A). By using a relative quantification of single nucleotide polymorphism (SNP) method to detect the NA genotype at codon 275 (CAC or TAC), we found that the virus population in the co-inoculated ferret's nasal washes remained mixed but was predominantly a wild-type (oseltamivir-sensitive) population (Figure 3B). The proportion of wild-type virus in the nasal wash increased progressively, from 75% on day 1 p.i., to almost 100% on day 6 p.i. (Figure 3B). Two of 2 ferrets placed in direct contact with the co-inoculated ferret were infected through transmission (Figure 3A). SNP analysis of their nasal wash samples showed only wild-type virus (Figure 3B). In summary, the oseltamivir-sensitive A/DM/524/09 virus possessed greater growth capability in the upper respiratory tract than did resistant A/DM/528/09 virus and thus had an advantage in direct-contact transmission.

## Discussion

This study is the first, to our knowledge, to demonstrate the inefficient respiratory droplet transmission of an oseltamivir-resistant H275Y mutant of H1N1/2009 in ferrets, which are an established animal model of the pathogenesis and transmission of human influenza viruses. The oseltamivir-resistant mutant virus retained efficient transmission only by direct contact, whereas the oseltamivir-sensitive pandemic virus was efficiently transmitted by both routes. These results show that the transmissibility of the oseltamivir-resistant H1N1/2009 influenza virus had been altered. We suggest that the lower fitness of oseltamivir-resistant variant within the host along with its reduced NA enzyme efficiency and delayed growth of the H275Y mutant virus *in vitro* may at least in part explain its impaired transmission among ferrets.

There are limited experimental data about the routes of transmission of oseltamivir-resistant influenza viruses. The two natural routes of influenza virus transmission, direct contact with fomites and respiratory droplets (aerosol and larger droplets [33]), are not mutually exclusive. Therefore, the transmissibility of influenza virus via both routes must be investigated if the results are to be clinically relevant. In the earliest studies, oseltamivir-resistant H3N2 (R292K NA mutant) and H1N1 (H275Y NA mutant) variants exhibited severely compromised replication and virulence both *in vitro* and *in vivo* [34], [35] and were therefore thought unlikely to be of clinical consequence. In a subsequent study, an R292K mutant of H3N2 virus was not transmitted by direct contact among ferrets [28]. Under similar conditions, the transmission of an E119V mutant of H3N2 virus and an H275Y mutant of A/New Caledonia/20/99-like (H1N1) virus by direct contact required a higher dose of inoculum than transmission of the wild-type viruses, and it occurred more slowly [27]. However, none of these studies assessed both routes of transmission. The only study to date that has evaluated both routes of transmission of oseltamivir-resistant virus showed that recombinant resistant H3N2 viruses with either the E119V or the E119V+I222V NA mutation were transmitted efficiently by direct contact but not by respiratory droplets among guinea pigs [26]. Our study is a latest addition to the previous data by comparing a highly matched pair of H1N1/2009 viruses and by assessing the transmissibility of resistant viruses via two routes in ferrets.

The reduced transmissibility of the oseltamivir-resistant H1N1 viruses could be explained by a number of factors [33], [36], [37]. First, host physical exposure to virus is directly affected by the quantity of virus shed into the environment. In our study, inoculated donor ferrets shed comparable quantities of both viruses, which indicated potential comparable environmental contamination in the restricted space of cages; therefore, it is unlikely that transmission was affected by the level of donor viral shedding. Other host variables such as the extent of inflammation could affect the amount and size of upper respiratory secretions thus the release of infectious respiratory droplets. For example, sneezing, a common host symptom believed to mediate viral transmission, was observed only at later stages in the ferret inoculated with resistant virus, when inflammation was more severe but virus shedding had declined greatly. Second, efficient transmission to a naive host requires not only viral exposure but also successful viral invasion, effective replication and simultaneous evasion of the first line of host innate immunity [38]. Our results showed a significant initial growth delay in two cell lines of the oseltamivir-resistant virus. This growth delay could be caused by delayed release of progeny virions from the host cell surface due to reduced NA enzyme efficiency observed in the resistant virus. Such a delay would not affect the final virus yield in cell lines, but in the respiratory tract of ferrets it could allow the host's first-line innate immune defense (e.g., macrophages or neutrophils) sufficient time to clear the virus. The NA enzyme also facilitates virus binding, entry, and spread within the host by removing terminal sialic-acid residues from mucus and preventing virion self-aggregation [39], and therefore the NA mutation could have affected viral penetration into the host respiratory tract. The slightly reduced (not severely impaired) NA enzyme function and delayed viral growth of the H275Y mutant may have been more crucial in recipient ferrets that acquired virus from environment via natural routes than in donor ferrets inoculated with a high dose of virus, as we observed delayed viral shedding or inefficient transmission in the recipient ferrets, but not in the inoculated donor ferret.

Although the transmissibility of the oseltamivir-resistant H1N1/2009 virus was reduced by the H275Y NA mutation, the severity and course of disease was similar to that caused by oseltamivir-sensitive H1N1/2009 virus in both inoculated and direct-contact ferrets, with no apparent attenuation of clinical signs. In inoculated ferrets, the viruses showed comparable replication in the upper respiratory tract and caused comparable clinical signs, including weight loss and inflammation. However, one caveat to ferret model has been noticed that high inoculation dose may mask the differential viral replication and clinical signs for different viruses [40]. In the direct-contact ferrets, which acquired virus though natural routes, the shedding of resistant virus peaked later than the shedding of susceptible virus, but the duration of shedding and the severity of disease was not compromised when compared with sensitive virus. Therefore, the H275Y mutant of pandemic H1N1/2009 virus is likely to be of clinical consequence in humans.

The fitness of a virus describes its relative ability to produce infectious progeny in a host [41]. Competitive growth assay by co-infection is a method of evaluating the growth fitness of two viruses [41], [42]. In the present study, we inoculated a ferret with equal doses of oseltamivir-sensitive and -resistant H1N1/2009 viruses to compare their relative growth fitness within the host. The mixed virus population in the nasal wash was analyzed at different days p.i. to determine which viral genotype predominated. To bypass the time- and labor-intensive process of cloning the desired genes from the mixed populations and choosing an arbitrary number of clones for genotypic analysis, we used a new method, relative quantification of SNP, to determine the ratio of wild-type to mutant populations. This method showed high reproducibility in genotyping HIV protease gene [42]. Our study is the first to use this method to genotypically analyze influenza viruses. For the H1N1/2009 influenza viruses, we designed a specific probe to detect the first nucleotide of codon 275 of the NA gene, where a single C-to-T substitution causes an H-to-Y amino acid substitution. Our results showed that the oseltamivir-resistant mutant H1N1/2009 virus possessed less growth fitness than the sensitive H1N1/2009 virus in the ferret upper respiratory tract. At least partly for that reason, only wild-type H1N1/2009 virus was transmitted to the direct-contact ferrets. The competitive transmission advantage of wild-type H1N1/2009 virus should be confirmed by other types of experiments.

In summary, our study determined the comparative transmissibility of a pair of naturally circulating oseltamivir-sensitive and oseltamivir-resistant H1N1/2009 viruses. This information from this study could be useful in assessing the clinical relevance of contemporary pandemic viruses, considering the extensive use of oseltamivir during this pandemic. The H275Y mutant of H1N1/2009 used in this study was the first oseltamivir-resistant H1N1/2009 isolate from a patient on oseltamivir prophylaxis. As this study was undertaken, additional H275Y mutants of H1N1/2009 viruses have emerged in the absence of oseltamivir use [21]–[25]. The emergence of these viruses should raise concerns as to whether resistant H1N1/2009 viruses will acquire significantly greater fitness and spread worldwide as did the naturally resistant H1N1 viruses during the 2007–2008 season. Further studies of these newly isolated H275Y mutants of H1N1/2009 viruses are warranted to determine whether they have acquired additional changes.

## Materials and Methods

### Ethics statement

All animal experiments with H1N1 influenza viruses were performed in biosafety level 3+ facilities at St. Jude Children's Research Hospital (St. Jude; Memphis, TN, USA), were approved by the St. Jude Animal Care and Use Committee, and complied with the policies of the National Institutes of Health and the Animal Welfare Act.

### Viruses and cells

A/Denmark/524/09 (H1N1) influenza virus (A/DM/524/09) and an oseltamivir-resistant A/Denmark/528/09 (H1N1) virus (A/DM/528/09) were provided by Statens Serum Institute, Copenhagen, Denmark. The resistant virus was isolated from the tthroat swab of a patient who had influenza-like symptoms and received post-exposure oseltamivir prophylaxis (75 mg once daily) [30]. A/Brisbane/59/07 (H1N1) influenza virus (A/BR/59/07) was provided by U.S. Centers for Disease Control and Prevention. Stocks of H1N1 viruses were prepared in Madin-Darby canine kidney (MDCK) cells (ATCC, Manassas, VA) and grown in minimal essential medium (MEM) supplemented with 5% fetal bovine serum, 5 mM L-glutamine, 0.2% sodium bicarbonate, 100 U/ml penicillin, 100 µg/ml streptomycin sulfate, and 100 µg/ml kanamycin sulfate in a humidified atmosphere of 5% CO_2_. All strains of virus underwent a limited number of passages in MDCK cells to maintain their original properties. MDCK cells transfected with cDNA encoding human 2,6-sialyltransferase (MDCK-SIAT1 cells) were maintained as described previously [32].

### Compounds

The NA inhibitors oseltamivir carboxylate ([*3R*, *4R*, *5S*]-4-acetamido-5-amino-3-[1-ethylpropoxy]-1-cyclohexene-1-carboxylic acid) and zanamivir (4-guanidino-Neu5Ac2en) were provided by Hoffmann-La Roche, Ltd. (Basel, Switzerland). The compounds were dissolved in distilled water and aliquots were stored at −20°C until the time of use.

### Infectivity of influenza viruses

The 50% tissue culture infectious dose (TCID_50_) was determined in MDCK cells. The cells were infected with serial log dilutions of the stock viruses, incubated for 1 h at 37°C, washed, and overlaid with infection medium (MEM with 0.3% BSA and 1 µg/ml TPCK-trypsin). Infection of cells was determined by hemagglutination assay (HI) after incubation for 3 d at 37°C, and TCID_50_ was calculated by the Reed-Muench method [43].

### Replication kinetics

Single-step growth curves were generated for influenza viruses in MDCK cells or MDCK-SIAT1 cells. Confluent cell monolayers were infected with viruses at a multiplicity of infection (MOI) of ∼2.0 PFU/cell. After incubation, the cells were washed with 0.9% aqueous NaCl solution (pH 2.2) to remove free infectious virus particles and then were washed twice with phosphate-buffered saline (PBS) to adjust the pH. Supernatants were collected 2, 4, 6, 8, 10 and 12 h p.i. and stored at −70°C for titration. To generate multi-step growth curves, MDCK cells or MDCK-SIAT1 cells were infected with viruses at a MOI of 0.001 PFU/cell. Supernatants were collected 12, 24, 36, 48, 60 and 72 h p.i. and stored at −70°C for titration in the same cell line.

### Plaque assay in MDCK and MDCK-SIAT cells

Confluent MDCK or MDCK-SIAT cells were incubated for 1 h at 37°C with 10-fold serial dilutions of virus in 1 ml of infection medium. The cells were then washed and overlaid with freshly prepared MEM containing 0.3% BSA, 0.9% bacto-agar, and 1 µg/ml TPCK trypsin. The plaques were visualized after incubation at 37°C for 3 d by staining with 0.1% crystal violet solution containing 10% formaldehyde.

### Virus susceptibility to NA inhibitors *in vitro*

A modified fluorometric assay using the fluorogenic substrate 2′-(4-methylumbelliferyl)-α-D-N-acetylneuraminic acid (MUNANA) (Sigma-Aldrich) was used to determine viral NA activity [44]. The fluorescence of the released 4-methylumbelliferone was measured in a Synergy 2 multi-mode microplate reader (BioTek) using excitation and emission wavelengths of 360 and 460 nm, respectively. The drug concentration required to inhibit 50% of the NA enzymatic activity (IC_50_) was determined by plotting the percent inhibition of NA activity as a function of compound concentration calculated in the GraphPad Prism 4 software from the inhibitor-response curve. The NA inhibitor–sensitive A/Fukui/20/04 (H3N2) influenza virus was included in every plate for comparison.

### NA enzyme kinetics

All H1N1 viruses were standardized to an equivalent dose of 10^6.0^ PFU/ml. We measured NA enzyme kinetics at pH 6.5 with 33 mM 2-(N-Morpholino) ethanesulfonic acid hydrate (MES; Sigma-Aldrich), 4 mM CaCl_2_, and MUNANA with a final substrate concentration of 0 to 400 µM. The reaction was conducted at 37°C in a total volume of 50 µl, and the fluorescence of released 4-methylumbelliferone was measured every 60 sec for 60 min in a Synergy 2 multi-mode microplate reader (BioTek) using excitation and emission wavelengths of 360 and 460 nm, respectively. The Km and Vmax were calculated by fitting the data to the appropriate Michaelis-Menten equations by using nonlinear regression in the GraphPad Prism 4 software. The A/PR/8/34 (H1N1) influenza virus was included for comparison in all assays.

### Transmission experiments in ferrets

Young adult ferrets (4–5 months of age) were obtained from the ferret breeding program at St. Jude Children's Research Hospital. All ferrets were seronegative for influenza A H1N1 and H3N2 viruses and for influenza B viruses. Ferrets were housed in the isolators in ABSL3+ facilities and monitored for 3–5 days to establish baseline body temperature and overall health. Donor ferrets were initially housed separately from contact ferrets. The donor ferrets were lightly anesthetized with isoflurane and inoculated with 10^6^ TCID_50_ of A/DM/524/09, A/DM/528/09 virus in 1.0 ml sterile PBS. One donor ferret was inoculated with 10^6^ TCID_50_ of a mixture of A/DM/524/09 and A/DM/528/09 viruses (1∶1 infectivity ratio). After the donor ferrets were confirmed to shed virus on day 2 p.i. by the Directigen Flu A+B quick test (BD, Franklin Lakes, NJ), each was then housed in the same cage with 2 naïve direct-contact ferrets. Two additional recipient ferrets were placed in an adjacent cage isolated from the donor's cage by a two layers of wire mesh (∼5 cm apart) that prevented physical contact but allowed the passage of respiratory droplets. A Borazine gun (Zero Toys, Concord, MA ) was used to ensure non-directional air flow inside the isolator. The donor and recipient ferrets were housed together since day 2 p.i until day 21 p.i. Ferret weight and temperature were recorded daily for 21 days. Body temperature was measured by subcutaneous implantable temperature transponders (Bio Medic Data Systems Inc, Seaford, DE).

### Collection and titration of nasal wash samples

Nasal washes were collected from donors and recipients on days 1, 2, 4, 6, 8, 10, 12, and 14 p.i. by flushing both nostrils with 1.0 ml PBS, and TCID_50_ titers were determined in MDCK cells. Inflammatory cell counts were determined as described previously [45]. Briefly, the nasal washes were centrifuged at 2000 rpm for 5 min. The pellet was resuspended in PBS, and the total cell number was counted in a hemacytometer under light microscopy. Inflammation was defined as a cell count ≥10 times the baseline count determined before the inoculation or exposure.

### Serologic tests

Serum samples were collected from ferrets 3 weeks after virus inoculation, treated with receptor-destroying enzyme, heat-inactivated at 56°C for 30 min, and tested by HI assay with 0.5% packed chicken red blood cells (CRBC) as described previously [46].

### Virus sequence analysis

Viral RNA was isolated from ferret nasal washes by using the RNeasy Mini kit (Qiagen, Valencia, CA). Samples were reverse-transcribed and analyzed by PCR using primers specific for the NA gene segment, as described previously [47]. Sequencing was performed by the Hartwell Center for Bioinformatics and Biotechnology at St. Jude Children's Research Hospital. The DNA template was sequenced by using rhodamine or dRhodamine dye terminator cycle-sequencing Ready Reaction kits with AmpliTaq DNA polymerase FS (Perkin-Elmer, Applied Biosystems, Inc., Foster City, CA) and synthetic oligonucleotides. Samples were analyzed in a Perkin-Elmer Applied Biosystems DNA sequencer (model 373 or 377). DNA sequences were completed and edited by using the Lasergene sequence analysis software package (DNASTAR, Madison, WI). The alignment of NA and HA for multiple sequences was conducted by BioEdit software (Tom Hall Ibis Therapeutics, Carlsbad, CA).

### Relative quantification of single nucleotide polymorphism (SNP)

The relative quantification of SNP assay was performed as described previously [42], with slight modification. Briefly, an NA fragment (nucleotide 673 to 1034) containing the codon at NA 275 position was amplified by RT-PCR. Primers were 5′-AGAACACAAGAGTCTGAATGTG-3′ and 5′-CCATTTGCTCCATTAGACGATACT-3′. Single nucleotide primer extension was performed using a SNaPshot kit (ABI) per the manufacturer's protocol. The reaction consisted of 2.5 µl SNaPshot Reaction Mix, 3 µl RT-PCR product, and 0.2 µmol/L of the extension primer in a 5 µl final reaction volume. The extension primer, 5′-CAGTCGAAATGAATGCCCCTAATTAT-3′, was synthesized by IDTDNA and used to detect the first nucleotide of NA 275 codon. After the SNaPshot reaction, a unit of shrimp alkaline phosphatase (USB) was added to remove 5′ phosphoryl groups of unincorporated dideoxynucleotide substrates as directed by manufacturer's protocol. One µl of the SNaPshot products was mixed with deionized formamide and LIZ120 (ABI) size standard and was injected into the ABI 3730xl capillary electrophoresis instrument (ABI) per the manufacturer's protocol. Data were analyzed by using ABI GeneMapper software. Serially diluted DNA template (35 ng/µl to 0.02 ng/µl) from each genotype was used for signal standardization. Spike-in samples were generated by using 11 different ratios of wild-type and mutant DNA fragments, e.g. 100% wild-type, 90% wild-type, etc. A good correlation was achieved between the spike-in ratios and ratios of fluorescence intensity values (R^2^ = 0.9877) (data not shown).

### Statistical analysis

The unpaired *t*-test or analysis of variance (ANOVA) was used for all comparisons.
